# Efficacy and safety of proton pump inhibitors versus vonoprazan in treatment of erosive esophagitis: A PRISMA-compliant systematic review and network meta-analysis

**DOI:** 10.1097/MD.0000000000031807

**Published:** 2022-11-25

**Authors:** Sensen Yang, Weishang Deng, Zeyu Xie, Jisheng Chen

**Affiliations:** a Key Specialty of Clinical Pharmacy, The First Affiliated Hospital of Guangdong Pharmaceutical University, Guangzhou, China.

**Keywords:** erosive esophagitis, proton-pump inhibitors, vonoprazan

## Abstract

Proton-pump inhibitors (PPIs) and vonoprazan are recommended as first-line therapies for erosive esophagitis (EE). However, it is uncertain how the magnitude of efficacy and safety of first-line therapy, the choice of individual PPIs or vonoprazan in the treatment of EE remains controversial. This study aimed to evaluate the efficacy and safety of vonoprazan and PPIs in healing esophageal mucosal injury in patients with EE.

**Methods:**

Relevant databases were searched to collect randomized controlled trials of proton pump inhibitors and vonoprazan in the treatment of reflux esophagitis up to December 2021. Studies on standard-dose PPIs or vonoprazan that were published in Chinese or English and assessed healing effects in EE were included in the analysis. Stata16.0 was used to conduct a network Meta-analysis to evaluate the efficacy and safety of the treatment.

**Results:**

A total of 41 literatures were included with 11,592 enrolled patients. For the endoscopic cure rate, all the PPIs and vonoprazan significantly improve compared to Placebo; Based on the surface under the cumulative ranking curve, Ilaprazole ranked first, followed by esomeprazole, vonoprazan, pantoprazole, lansoprazole, omeprazole, rabeprazole and placebo therapy ranked the last. For the rate of adverse events, there was no significant difference among all the PPIs, vonoprazan, and placebo.

**Conclusions:**

Ilaprazole, esomeprazole and vonoprazan have more advantages in mucosal erosion healing, there was no significant difference in the comparative safety among all interventions.

## 1. Introduction

Erosive esophagitis (EE) is classified as a type of gastroesophageal reflux disease (GERD), which generally involves changes in the barrier between the stomach and the esophagus, esophageal clearance dysfunction and weakened epithelial defense function, increased esophageal sensitivity.^[1,2]^ The corresponding symptoms or mucosal damage are caused by the reflux of gastric contents from the stomach into the esophagus, including heartburn, regurgitation, odynophagia, nausea, chest pain and coughing. Complications may deteriorate and even further develop into esophageal strictures, Barrett’s esophagus, and even esophageal adenocarcinoma without adequate treatment.^[3,4]^

Currently, the main treatments for EE include drug therapy, surgery, and lifestyle and dietary modifications, and drug therapy is one of the essential therapeutic methods. Notably, standard-dose vonoprazan and proton-pump inhibitors (PPIs) were recommended as first-line therapy for EE by guidelines.^[5–8]^ However, the choice of individual PPIs drug or vonoprazan for the treatment of EE is still controversial. Previous studies have compared PPIs and vonoprazan for Heartburn Symptoms in EE,^[9]^ but few studies have investigated the healing of esophageal mucosal injuries. Therefore, we conducted this study to evaluate the comparative efficacy and safety of vonoprazan and PPIs for healing esophageal mucosal injury in EE.

## 2. Materials and Methods

The study referred to the recommended method by the Cochrane Handbook for Systematic Reviews of Interventions,^[10]^ and reported according to the Preferred reporting items for systematic reviews and meta-analyses statement.^[11]^

### 2.1. Data sources and searches

PubMed, Embase, Cochrane Library, China National Knowledge Infrastructure, China Biology Medicine disc, VIP database, and the Wanfang database were searched to identify randomized controlled trials of PPIs and vonoprazan in the treatment of EE up to December 2021. The search strategy searched for PubMed can be found in Table S1 (Supplemental Digital Content, http://links.lww.com/MD/H938, which shows the search strategies).

### 2.2. Eligibility criteria and study selection

Two authors independently screened the title and abstract of citations for relevant literature. Any disagreement would consult another author and solve by discussion. The inclusion criteria were the followings:

*Patients*: patients with EE. The definition of EE: patients have the condition symptoms with the presence of esophageal abnormalities by endoscopy. Exclude refractory EE or resistance to previous PPIs treatment.

*Interventions and comparisons*: We included studies which had at least 2 of the following interventions: omeprazole 20 mg/d, lansoprazole 30 mg/d, pantoprazole 40 mg/d, rabeprazole 20 mg/d, esomeprazole 40 mg/d, ilaprazole 10 mg/d, vonoprazan 20 mg/d, and placebo.

*Outcomes*: Four to eight weeks endoscopic cure rate and rate of adverse events.

*Study design*: We included randomized clinical trials in Chinese or English.

### 2.3. Data extraction and quality assessment

We independently extracted relevant information, including study characteristics (year of publication, duration), population (sample size, age, male-to-female ratio, patient demographics), description of interventions (drug class, name, dose), and outcomes. The number of healed patients or the observed healing rate by endoscopy at 4 to 8 weeks and the number or rate of adverse events were extracted for each treatment group from each study. Intention-to-treat data were collected for all outcomes when possible; otherwise, Full Analysis Set data were collected. The risk of bias was assessed using the Cochrane Risk of Bias Tool for randomized clinical trials.^[10]^

### 2.4. Data synthesis and analysis

We evaluated assumptions of transitivity and consistency before conducting data synthesizing.^[12]^ For transitivity assumption, the study method and patient characters at baseline for each study were compared.^[13,14]^ For the consistency assumption, The overall inconsistency was tested by using global approach and local approach.^[15]^ When the global *P* value in the global approach was more than 0.05 and the inconsistency factor approaches the lower limit of 0 or 95% confidence interval (CI) includes 0 or near 0, we considered that the consistency is accepted.^[15–17]^ If there was good transitivity across the study and no significant inconsistency, we would conduct network meta-analyses with random model.^[18]^ The efficacy and safety of them were ranked by using the surface under the cumulative ranking curve (SUCRA).^[19,20]^ For the same outcome, the larger SUCRA and the higher the rank probability, the better efficacy and safety of the treatment regimen. Funnel plot was used to assess publication bias.^[19]^ Moreover, sensitivity analyses were conducted to examine the robustness of results by excluding studies with a high risk of bias.

All data analyses were conducted through STATA software (version 16; StataCorp LP, College Station, TX).

## 3. Results

### 3.1. Study characteristics and risk of bias

**Figure 1.**
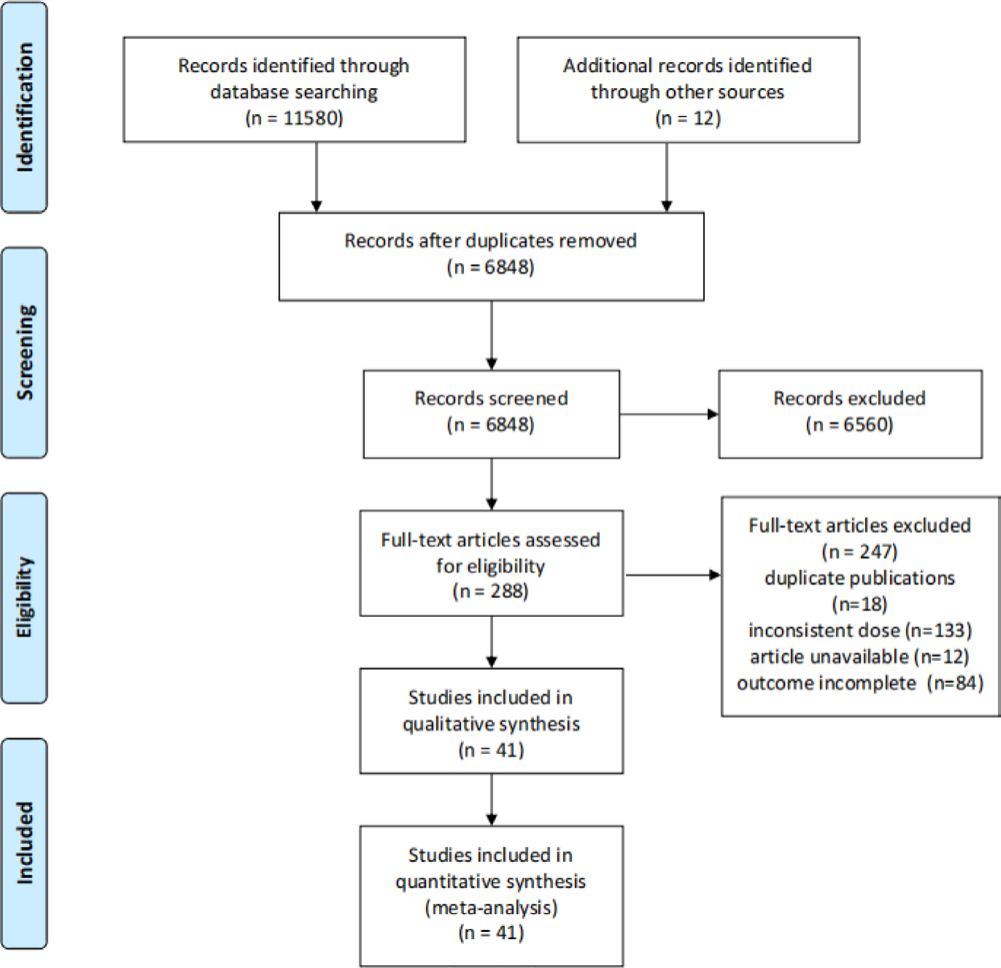
Flow chart of study selection.

**Figure 2.**
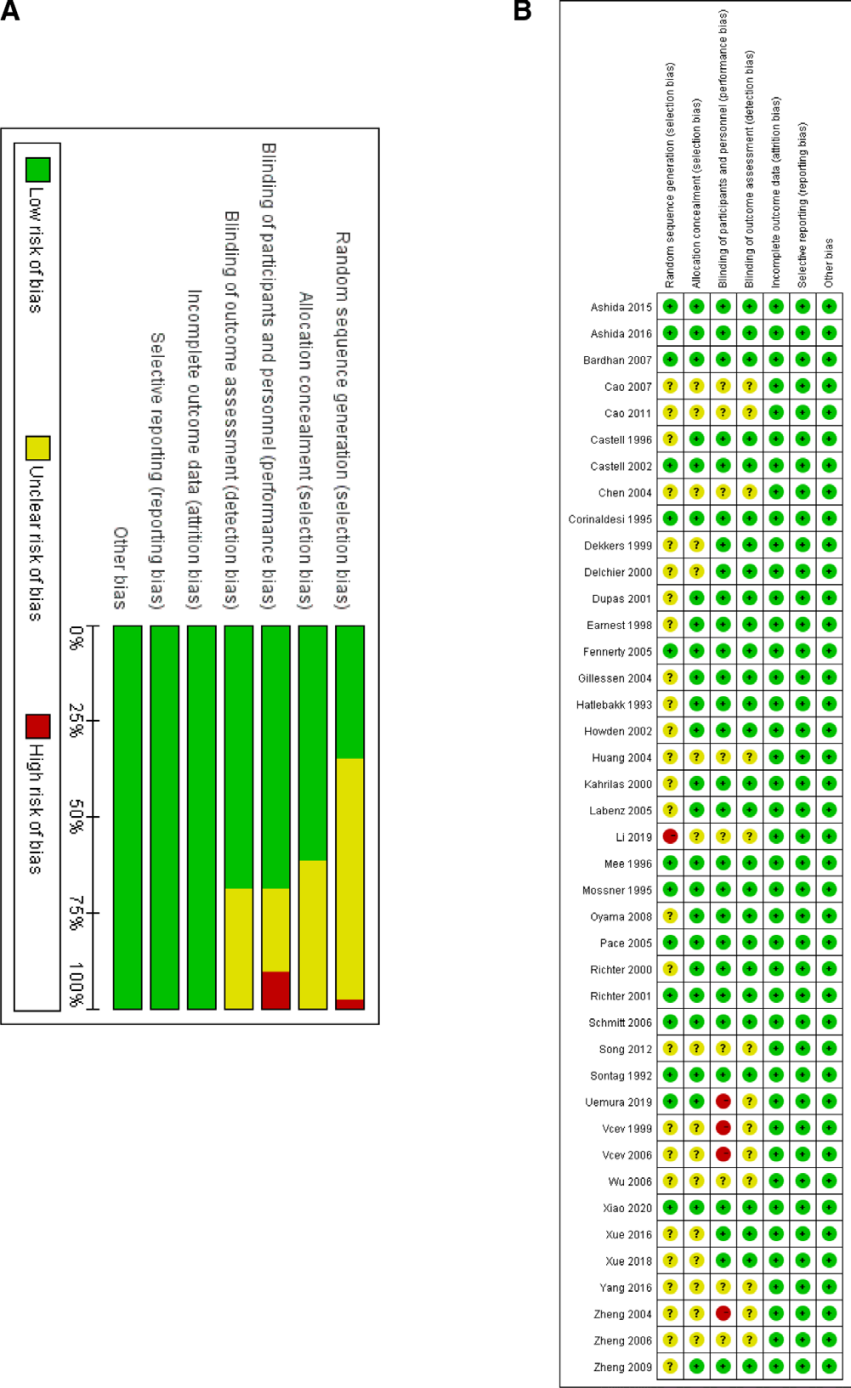
Risk of bias graph and summary. Green for low risk of bias (+), yellow for unclear risk of bias (?) and red for high risk of bias (-).

Figure 1 presented the election process, the systematic literature search identified 11,592 potential studies from the databases, and we imported citations into EndNoteX9. After excluding duplicates, we screened 6848 records by reading all titles and abstracts based on the selection criteria. And then, we reviewed full texts of 288 potentially relevant articles. Forty-one randomized controlled trials ultimately were included with 11,592 enrolled patients.^[21–61]^ The characteristics of the included studies were shown in Table S2 (Supplemental Digital Content, http://links.lww.com/MD/H939, which presents the characteristics of included studies). As shown in Figure 2, only 14 studies have a low risk of bias in random sequence generation.^[21,23,24,26,34,35,41,43,44,46,54,55,59,61]^ Twenty-five studies have used appropriate allocation concealment.^[21–27,31–37,41–44,46,50,51,54,55,59,61]^ Twenty-eight studies have a low risk of blinding of participants, personnel and outcome assessment.^[21–37,41–44,46,50,51,54–56,58,61]^ All 41 studies have complete data and none selectively reported the findings, and it was unclear whether other sources of bias existed.^[21–61]^

### 3.2. Rate of healing at 4 weeks

**Figure 3.**
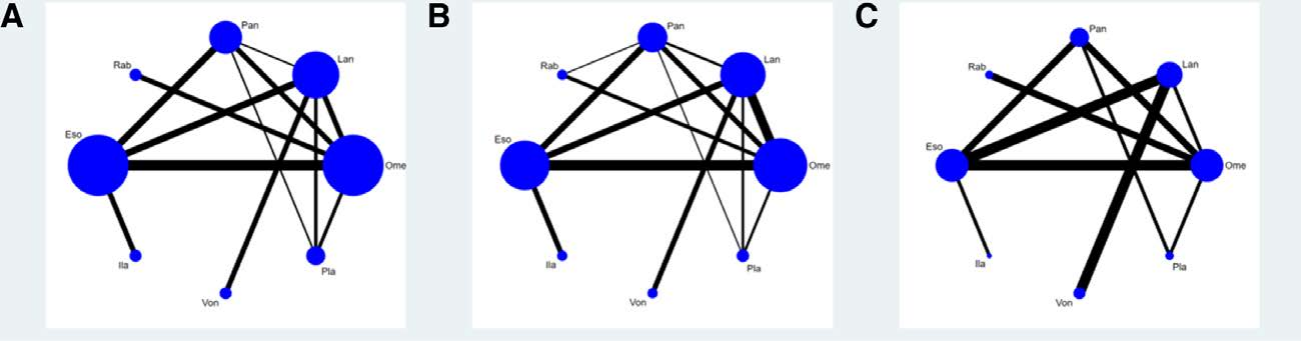
Network plots. Network plots for healing rates at 4 and 8 wk (A and B), network plot for adverse event rate (C). Ome, 20 mg/d; Pan, 40 mg/d; Lan, 30 mg/d; Rab, 20 mg/d; Ila, 10 mg/d; Eso, 40 mg/d; Von, 20 mg/d. Eso = esomeprazole, Ila = ilaprazole, Lan = lansoprazole, Ome = omeprazole, Pan = pantoprazole, PLA = placebo, Rab = rabeprazole, Von = vonoprazan.

Thirty-three studies have reported the endoscopic cure rate at 4 weeks, and 8 interventions were involved, including omeprazole, lansoprazole, pantoprazole, rabeprazole, esomeprazole, ilaprazole, and vonoprazan. The network plot was shown in Figure 3.

**Figure 4.**
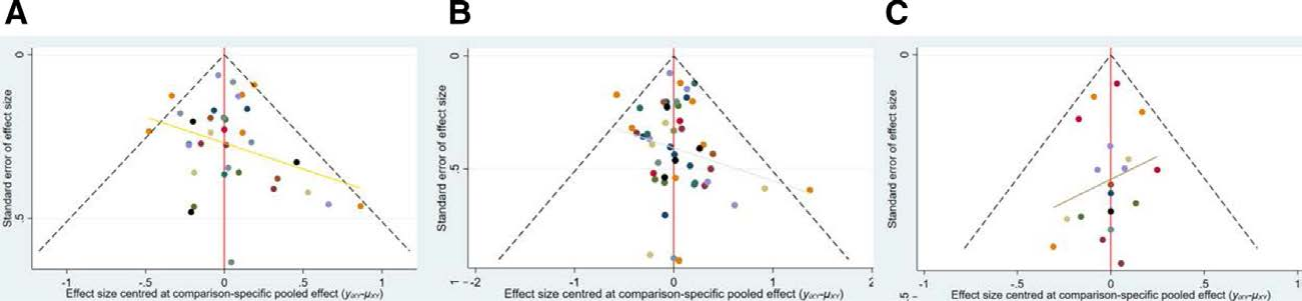
Funnel plots. Funnel plots for healing rates at 4 and 8 wk (A and B), funnel plot for adverse event rate (C).

The area of nodes indicates the number of studies included in the corresponding nodes, and the width of the lines connecting nodes suggested the number of relevant data. No inconsistency was detected through global (*P* = .7526) and loop-specific approach in the endoscopic cure rate at 4 weeks (Table 1). The results of the network meta-analysis (NMA) indicated that in terms of the 4-week endoscopic cure rate (Table 2): PPIs and vonoprazan significantly improved the 4-week healing rate compared to placebo. The cure rate of omeprazole was lower than esesomeprazole (odds ratio [OR] = 0.68, 95% CI = 0.59–0.79) and ilaprazole (OR = 0.72, 95% CI = 0.61–0.86), lansoprazole was lower than esomeprazole (OR = 0.72, 95% CI = 0.61–0.86), Pantoprazole was lower than esomeprazole (OR = 0.78, 95% CI = 0.65–0.94), Rabeprazole was lower than esomeprazole (OR = 0.61, 95% CI = 0.41–0.91). Table 3 presented the ranking of all interventions for the endoscopic cure rate at 4 weeks based on the SUCRA. Ilaprazole ranked first followed by esomeprazole, vonoprazan, pantoprazole, lansoprazole, omeprazole, rabeprazole and placebo therapy ranked the last. The funnel plot was indicated in Figure 4.

**Table 1:** Inconsistency factors of all comparisons.

Outcome	Cycle	IF and 95% CI	*P* value
4 wk healing rates	Ome-Pan-Pla	IF = 0.637, 95% CI (0.00, 1.51)	.153
	Ome-Lan-Pan	IF = 0.404, 95% CI (0.00, 1.01)	.190
	Lan-Pan-Pla	IF = 0.383, 95% CI (0.00, 1.29)	.410
	Lan-Pan-Eso	IF = 0.302, 95% CI (0.00, 0.82)	.250
	Ome-Pan-Eso	IF = 0.213, 95% CI (0.00, 0.77)	.455
	Ome-Lan-Pla	IF = 0.194, 95% CI (0.00, 0.78)	.518
	Ome-Lan-Eso	IF = 0.086, 95% CI (0.00, 0.58)	.736
8 wk healing rates	Ome-Pan-Rab	IF = 1.017, 95% CI (0.00, 2.85)	.277
	Ome-Pan-Eso	IF = 0.541, 95% CI (0.00, 1.23)	.124
	Ome-Lan-Eso	IF = 0.417, 95% CI (0.00, 0.88)	.077
	Ome-Lan-Pan	IF = 0.404, 95% CI (0.00, 1.07)	.237
	Lan-Pan-Eso	IF = 0.303, 95% CI (0.00, 0.95)	.356
	Ome-Pan-Pla	IF = 0.117, 95% CI (0.00, 0.96)	.785
	Lan-Pan-Pla	IF = 0.104, 95% CI (0.00, 1.00)	.820
	Ome-Lan-Pla	IF = 0.224, 95% CI (0.00, 0.80)	.448
Adverse event rates	Ome-Pan-Von	IF = 0.414, 95% CI (0.00, 1.46)	.437
	Ome-Pan-Eso	IF = 0.387, 95% CI (0.00, 1.12)	.302
	Ome-Lan-Eso	IF = 0.173, 95% CI (0.00, 0.83)	.608

CI = confidence interval; Eso = esomeprazole, 40 mg/d; IF = inconsistency factor; Ila = ilaprazole, 10 mg/d; Lan = lansoprazole, 30 mg/d; Ome = omeprazole, 20 mg/d; Pan = pantoprazole, 40 mg/d; PLA = placebo; Rab = rabeprazole, 20 mg/d; Von = vonoprazan 20 mg/d.

**Table 2:** Results of network meta-analyses for the rate of healing at 4 and 8 wk.

OR (95% CI)							
Ome	1.17 (0.94, 1.45)	1.31 (1.01, 1.69)*	0.84 (0.54, 1.32)	1.56 (1.29, 1.89)*	1.73 (1.11, 2.71)*	1.52 (0.91, 2.55)	0.09 (0.06, 0.13)*
0.94 (0.78, 1.14)	Lan	1.12 (0.84, 1.48)	0.72 (0.44, 1.19)	1.34 (1.08, 1.66)*	1.48 (0.94, 2.34)	1.30 (0.81, 2.09)	0.08 (0.05, 0.11)*
0.87 (0.71, 1.08)	0.93 (0.74, 1.17)	Pan	0.64 (0.39, 1.07)	1.20 (0.94, 1.51)	1.33 (0.83, 2.12)	1.16 (0.67, 2.01)	0.07 (0.05, 0.10)*
1.13 (0.78, 1.64)	1.19 (0.79, 1.81)	1.29 (0.84, 1.97)	Rab	1.86 (1.14, 3.02)*	2.06 (1.10, 3.87)*	1.81 (0.91, 3.58)	0.11 (0.06, 0.19)*
0.68 (0.59, 0.79)*	0.72 (0.61, 0.86)*	0.78 (0.65, 0.94)*	0.61 (0.41, 0.91)*	Eso	1.11 (0.74, 1.66)	0.97 (0.58, 1.63)	0.06 (0.04, 0.08)*
0.63 (0.42, 0.94)*	0.67 (0.44, 1.01)	0.72 (0.48, 1.09)	0.56 (0.32, 0.97)*	0.92 (0.64, 1.34)	Ila	0.88 (0.46, 1.69)	0.05 (0.03, 0.09)*
0.74 (0.48, 1.14)	0.78 (0.53, 1.16)	0.84 (0.53, 1.33)	0.65 (0.37, 1.16)	1.08 (0.70, 1.66)	1.17 (0.66, 2.06)	Von	0.06 (0.03, 0.11)*
9.33 (6.65, 13.11)*	9.90 (7.07, 13.86)*	10.67 (7.42, 15.33)*	8.29 (5.01, 13.72)*	13.66 (9.68, 19.29)*	14.78 (8.89, 24.56)*	12.67 (7.55, 21.29)*	Pla

Results of 4 wk healing rates were listed in right upper triangles and results of 8 wk healing rates were listed in left lower triangles.

CI = confidence interval; Eso = esomeprazole, 40 mg/d; Ila = ilaprazole, 10 mg/d; Lan = lansoprazole, 30 mg/d; Ome = omeprazole, 20 mg/d; OR = odds ratio; Pan = pantoprazole, 40 mg/d; PLA = placebo; Rab = rabeprazole, 20 mg/d; Von = vonoprazan 20 mg/d.

* Significant difference between two treatments.

**Table 3:** The results of SUCRA.

Treatment	4 wk healing rates			8 wk healing rates			Adverse event rate		
	SUCRA	Pr. Best	MeanRank		Pr.Best	MeanRank	SUCRA	Pr. Best	MeanRank
Ome	31.6	0.0	5.8	27.7	0.0	6.1	65.9	12.1	3.4
Lan	41.7	0.0	5.1	45.7	0.0	4.8	57.4	7.3	4.0
Pan	54.3	0.1	4.2	59.9	1.6	3.8	47.5	10.2	4.7
Rab	24.4	0.3	6.3	20.5	0.2	6.6	54.3	26.2	4.2
Eso	85.3	21.4	2.0	82.2	14.3	2.2	57.9	3.7	3.9
Ila	88.8	55.1	1.8	88.1	53.9	1.8	45.2	24.4	4.8
Von	73.9	23.2	2.8	75.9	30.0	2.7	41.0	7.4	5.1
Pla	0.0	0.0	8.0	0.0	0.0	8.0	30.7	8.8	5.8

Eso = esomeprazole, 40 mg/d; Ila = ilaprazole, 10 mg/d; Lan = lansoprazole, 30 mg/d; Ome = omeprazole, 20 mg/d; Pan = pantoprazole, 40 mg/d; PLA = placebo; Pr. Best = probability of being the best; Rab = rabeprazole, 20 mg/d; SUCRA = the surface under the cumulative ranking curve; Von = vonoprazan 20 mg/d.

### 3.3. Rate of healing at 8 weeks

Forty-one studies have reported the endoscopic cure rate at 8 weeks. No inconsistency was detected through global (*P* = .4973) and loop-specific approach (Table 1).

The results of the NMA indicated that in terms of 8-week endoscopic cur-e rate (Table 2): pantoprazole is higher than omeprazole (OR = 1.31, 95% CI = 1.01–1.69), esomeprazole was higher than omeprazole (OR = 1.56, 95% CI = 1.29–1.89), lansoprazole (OR = 1.34, 95% CI = 1.08–1.66), and rebeprazole (OR = 1.86, 95% CI = 1.14–3.02). Ilaprazole was higher than omeprazole (OR = 1.73, 95% CI = 1.11–2.71) and rabeprazole (OR = 2.06, 95% CI  = 1.10–3.87). The 8-week endoscopic cure rat-e of PPIs and vonoprazan were significantly higher than the placebo.

Table 3 presented the ranking of all interventions for the endoscopic cure rate at 8 weeks based on the SUCRA. Ilaprazole ranked first followed by esomeprazole, vonoprazan, pantoprazole, lansoprazole, omeprazole, rabeprazole and placebo therapy ranked the last. The funnel plot was indicated in Figure 4.

### 3.4. Rate of adverse events

Adverse event rates were reported in 20 studies involving all 8 interventions, forming 5 loops. No inconsistency was detected through global (*P* = .1909) approach. However, the result of loop-specific approach indicated that cyclo lansoprazole- pantoprazole-esomeprazole presented inconsistency (Table S3, Supplemental Digital Content, http://links.lww.com/MD/H940, which presents the results of the loop-specific approach to adverse event rate). Therefore, we tested for inconsistency based on the node-splitting method (Table S4, Supplemental Digital Content, http://links.lww.com/MD/H941, which presents the results of node-splitting method on adverse event rate). The results showed that the inconsistency might come from the study by Dupas et al,^[33]^ which was the only head-to-head study of lansoprazole and pantoprazole reporting adverse event rates. There may be a small sample effect, so this study was excluded. The remaining 19 literature were analyzed, and the evidence relationship diagram was shown in Figure 3. No inconsistency was detected through global (*P* = .6737) and loop-specific approach (Table 1). As shown in Table 4, there was no significant difference in the incidence of adverse events among all the PPIs, vonoprazan, and placebo. The results of SUCRA (Table 3) indicated that the relative ranking safety was: omeprazole ranked first followed by esomeprazole, lansoprazole, rabeprazole, pantoprazole, ilaprazole, vonoprazan and placebo. The funnel plot was indicated in Figure 4.

**Table 4:** Results of network meta-analyses for adverse event rate.

OR (95% CI)							
Ome							
0.97 (0.78, 1.21)	Lan						
0.93 (0.68, 1.28)	0.96 (0.69, 1.34)	Pan					
0.97 (0.61, 1.52)	0.99 (0.60, 1.64)	1.04 (0.60, 1.80)	Rab				
0.97 (0.83, 1.14)	1.00 (0.87, 1.16)	1.05 (0.77, 1.41)	1.01 (0.62, 1.63)	Eso			
0.90 (0.46, 1.76)	0.93 (0.48, 1.80)	0.97 (0.47, 1.98)	0.93 (0.41, 2.09)	0.92 (0.48, 1.77)	Ila		
0.91 (0.65, 1.27)	0.93 (0.72, 1.21)	0.98 (0.64, 1.49)	0.94 (0.53, 1.66)	0.93 (0.69, 1.26)	1.01 (0.49, 2.06)	Von	
0.82 (0.51, 1.32)	0.84 (0.51, 1.40)	0.88 (0.56, 1.38)	0.85 (0.44, 1.64)	0.84 (0.52, 1.36)	0.91 (0.41, 2.05)	0.90 (0.51, 1.59)	Pla

CI = confidence interval; Eso = esomeprazole, 40 mg/d; Ila = ilaprazole, 10 mg/d; Lan = lansoprazole, 30 mg/d; Ome = omeprazole, 20 mg/d; OR = odds ratio; Pan = pantoprazole, 40 mg/d; PLA = placebo; Rab = rabeprazole, 20 mg/d; Von = vonoprazan 20 mg/d.

### 3.5. Sensitivity analysis

The results of sensitivity analyses based on data from studies with a low risk of bias, indicating the robustness of the results (Tables S5 and S6, Supplemental Digital Content, http://links.lww.com/MD/H942, which present the sensitivity analyses results of subgroup analysis of primary outcomes).

## 4. Discussion

In this study, we performed a NMA to evaluate the comparative efficacy and safety of PPIs and vonoprazan for treating EE. Moreover, we have ranked order based on efficacy and safety.

The results indicated that all the PPIs and vonoprazan significantly improved the endoscopic cure rate compared to placebo, endoscopic cure rates were higher at 8 weeks than 4 weeks for all interventions. Notably, in terms of endoscopic cure rates at 4 and 8 weeks, the NMA results showed some statistical differences between partial interventions, but frequently the 95% CI are relatively close to the 1. For example, statistically significant results were observed for pantoprazole compared with omeprazole in 8-week healing rate, but the effect was almost borderline. We should be cautious in interpreting the results and avoid exaggerating intervention differences.

According to SUCRA rank results, ilaprazole, esomeprazole, and vonoprazan might have more advantages in mucosal erosion healing. The advantages could be interpreted by its property of acid control.^[62]^ Ilaprazole, esomeprazole and vonoprazan produced relatively good effects on acid suppression and maintained PH > 4 for a relatively long time, which could promote mucosal healing.^[63–66]^

Ilaprazole is a new drug approved in China which was not included in previous studies. The results of our study are consistent with a NMA of 25 articles by Li et al,^[62]^ showed that standard-dose esomeprazole was superior in mucosal erosion healing to other PPIs. Miyazaki’s analysis indicated that the GERD-healing effect of vonoprazan is higher than rabeprazole but not higher than other PPIs,^[67]^ which was inconsistent with our study. On the 1 hand, this could be attributed to the dose of esomeprazole in their studies was 20 mg/d. On the other hand, the included participants in their studies were GERD, which involved non-EE.

For the rate of adverse events, our results were totally the same as the previous studies,^[62,68]^ there was no significant difference among all interventions.

There are several limitations to this study. Firstly, literature-based meta-analyses include heterogeneity and bias based on each study. Therefore, we performed sensitivity analyses to examine the robustness of the results by excluding studies with a high risk of bias. Secondly, the outcomes of the majority include studies have not been reported according to severity grading under endoscopy, and the grading systems differed among studies so that we can not assess the efficacy for patients with different severity of EE. Finally, most of the included studies have not reported the results of patients with CYP2C19 genotype and Helicobacter pylori infection, which meant we could not evaluate the impact of genetic polymorphism and Helicobacter pylori infection on drug therapy for EE.

Therefore, further high-quality, large-sample, and comprehensive clinical trials of vonoprazan and PPIs are required to confirm the efficacy and safety of the treatment of EE.

## 5. Conclusion

We have conducted NMA to analysis the efficacy and safety of vonoprazan and PPIs in EE. The results suggested that ilaprazole, esomeprazole, and vonoprazan have more advantages compared with other PPIs in mucosal erosion healing, while the safety of vonoprazan was similar to PPIs. To further confirm our conclusions, high-quality, large-sample and multi-center studies are required to provide more evidence.

## Author contributions

All authors contributed to the study conception and design. Project development: Jisheng Chen and Sensen Yang. Data collection or management: Sensen Yang and Weishang Deng. Data analysis and interpretation: Sensen Yang and Zeyu Xie. Manuscript writing: Sensen Yang and Weishang Deng. Manuscript editing: Jisheng Chen.

**Data curation:** Sensen Yang.

**Investigation:** Weishang Deng, Jisheng Chen.

**Methodology:** Sensen Yang.

**Resources:** Weishang Deng.

**Software:** Sensen Yang.

**Validation:** Zeyu Xie.

**Visualization:** Zeyu Xie.

**Writing – original draft:** Sensen Yang, Jisheng Chen.

**Writing – review & editing:** Jisheng Chen.

## Supplementary Material
