# Supplementary material for: Efficacy and safety of proton pump inhibitors versus vonoprazan in treatment of erosive esophagitis: A PRISMA-compliant systematic review and network meta-analysis
Source: Medicine (Baltimore). 2022 Nov 25;101(47):e31807. doi: 10.1097/MD.0000000000031807 (PMC9704910; doi:10.1097/MD.0000000000031807)
Supplement: Supplementary file 1 [file medi-101-e31807-s001.pdf]

Supplementary Material S1. Searching Strategy (PubMed)

#1 "Esophagitis, Peptic"[Mesh]
#2 (Esophagitides, Peptic[Title/Abstract]) OR (Peptic Esophagitides[Title/Abstract])) OR (Peptic Esophagitis[Title/Abstract]) OR (Esophagitis, Reflux[Title/Abstract]) OR (Esophagitides, Reflux[Title/Abstract]) OR (Reflux Esophagitides[Title/Abstract]) OR (Reflux Esophagitis[Title/Abstract]) OR (Erosive esophagitis[Title/Abstract])
#3 #1 OR #2
#4 "Proton Pump Inhibitors"[Mesh]
#5 Inhibitors, Proton Pump[Title/Abstract]) OR (Proton Pump Inhibitor[Title/Abstract])) OR (Inhibitor, Proton Pump[Title/Abstract]) OR (Pump Inhibitor, Proton[Title/Abstract])
#6 ("Omeprazole"[Mesh])
#7 (Prilosec[Title/Abstract]) OR (H 168-68[Title/Abstract]) OR (H 168 68[Title/Abstract]) OR (H 16868[Title/Abstract])
#8 "Rabeprazole"[Mesh]
#9 (2-((4-(3-methoxypropoxy)-3-methylpyridin-2-yl)methylsulfinyl)-1H-benzimidazole[Title/Abstract]) OR (Dexrabeprazole[Title/Abstract]) OR (E 3810[Title/Abstract]) OR (E3810[Title/Abstract]) OR (Pariet[Title/Abstract]) OR (Aciphex[Title/Abstract]) OR (LY-307640[Title/Abstract]) OR (LY 307640[Title/Abstract]) OR (LY307640[Title/Abstract])
#10 "Lansoprazole"[Mesh]
#11 (Lansoprazol[Title/Abstract]) OR (2-(((3-Methyl-4-(2,2,2-trifluoroethoxy)-2-pyridyl)methyl)sulfinyl)benzimidazole[Title/Abstract]) OR (Lansoprazoles[Title/Abstract]) OR (Ogastro[Title/Abstract]) OR (AG 1749[Title/Abstract]) OR (AG-1749[Title/Abstract]) OR (AG1749[Title/Abstract]) OR (Agopton[Title/Abstract]) OR (Bamalite[Title/Abstract]) OR (Lansol[Title/Abstract]) OR (Lanzor[Title/Abstract]) OR (Monolitum[Title/Abstract]) OR (Opiren[Title/Abstract]) OR (Prevacid[Title/Abstract]) OR (Pro Ulco[Title/Abstract]) OR (Promeco[Title/Abstract]) OR (Takepron[Title/Abstract]) OR (Ulpax[Title/Abstract]) OR (Zoton[Title/Abstract]) OR (Ogast[Title/Abstract]) OR (Prezal[Title/Abstract])
#12 "Esomeprazole"[Mesh]
#13 (Esomeprazole Sodium[Title/Abstract]) OR (Esomeprazole Strontium[Title/Abstract]) OR (Strontium, Esomeprazole[Title/Abstract]) OR (Esomeprazole Magnesium[Title/Abstract]) OR (Esomeprazole Potassium[Title/Abstract]) OR (Esomeprazole Strontium Anhydrous[Title/Abstract])) OR (Nexium[Title/Abstract])
#14 "Pantoprazole"[Mesh]
#15 (SKF-96022[Title/Abstract]) OR (SKF 96022[Title/Abstract]) OR (SKF96022[Title/Abstract]) OR (BY 1023[Title/Abstract]) OR (BY-1023[Title/Abstract]) OR (BY1023[Title/Abstract]) OR (Protonix[Title/Abstract])
#16 "ilaprazole" [Supplementary Concept]
#17 (IY 81149[Title/Abstract]) OR (IY81149[Title/Abstract]) OR (IY-81149[Title/Abstract])
#18 ("1-(5-(2-fluorophenyl)-1-(pyridin-3-ylsulfonyl)-1H-pyrrol-3-yl)-N-methylmethanamine" [Supplementary Concept])
#19 (Vonoprazan[Title/Abstract]) OR (TAK 438[Title/Abstract]) OR (TAK438[Title/Abstract]) OR (TAK-438[Title/Abstract])
#20 #4~#19 OR

#21 ("randomized controlled trial"[Publication Type] OR "controlled clinical trial"[Publication Type] OR "randomized"[Title/Abstract] OR "placebo"[Title/Abstract] OR "drug therapy"[MeSH Subheading] OR "randomly"[Title/Abstract] OR "trial"[Title/Abstract] OR "groups"[Title/Abstract]) NOT ("animals"[MeSH Terms] NOT "humans"[MeSH Terms])

#22 #3 AND #20 AND #21
