# Supplementary material for: Efficacy and safety of proton pump inhibitors versus vonoprazan in treatment of erosive esophagitis: A PRISMA-compliant systematic review and network meta-analysis
Source: Medicine (Baltimore). 2022 Nov 25;101(47):e31807. doi: 10.1097/MD.0000000000031807 (PMC9704910; doi:10.1097/MD.0000000000031807)
Supplement: Supplementary file 2 [file medi-101-e31807-s002.pdf]

Table S2. Characteristics of the included studies

Study	Treatment	Population	Age(years)	N(male)	Diagnosis level	outcome
Sontag,1992	Ome 20mg, qd	93	NA	NA	HD II-IV	1 2 3
	placebo	46	NA	NA		
Hatlebakk,1993	Lan 30mg, qd	116	54.3	77	Other 1–2	1 2 3
	Ome 20mg, qd	113	55.4	74		
Corinaldesi,1995	Pan 40mg, qd	120	50	78	SM 2-3	1 2 3
	Ome 20mg, qd	121	52	75		
Mossner,1995	Pan 40mg, qd	191	53	133	SM 2-3	1 2 3
	Ome 20mg, qd	95	55	66		
Castell,1996	Lan 30mg, qd	421	NA	NA	Other II-IV	1 2
	Ome 20mg, qd	431	NA	NA		
	placebo	213	NA	NA		
Mee,1996	Lan 30mg, qd	300	53.4	198	SM 1-4	1 2
	Ome 20mg, qd	304	52.4	204		
Earnest,1998	Lan 30mg, qd	66	NA	NA	Other II-IV	1 2
	placebo	71	NA	NA		
Dekkers,1999	Rab 20mg,qd	100	54±15.70	53	HD II-IV	1 2 3
	Ome 20mg, qd	102	52±15.56	73		
Vcev,1999	Pan 40mg, qd	60	NA	NA	SM 1-2	1 2
	Ome 20mg, qd	60	NA	NA		
Delchier,2000	Rab 20mg,qd	104	55±15.7	47	HD II-IV	1 2 3
	Ome 20mg, qd	103	53.0±15.1	40		
Kahrilas,2000	Eso 40mg,qd	654	44.8±13.0	384	LA A-D	1 2
	Ome 20mg, qd	650	46.5±13.5	399		
Richter,2000	Pan 40mg, qd	173	49.3±13.6	121	HD II-IV	1 2 3
	placebo	82	48.3±14.0	53		
Dupas,2001	Pan 40mg, qd	226	53.0±14.5	165	SM II-III	1 2 3
	Lan 30mg, qd	235	55.0±14.7	178		
Richter,2001	Eso 40mg,qd	1216	NA	722	LA I-IV	1 2 3
	Ome 20mg, qd	1209	NA	760		
Castell,2002	Eso 40mg,qd	2624	47.0±13.0	1504	LA A-D	1 2 3
	Lan 30mg, qd	2617	47.4±13.1	1501		
Howden,2002	Lan 30mg, qd	143	47.0±12.0	57	Other II-IV	1 2 3
	Eso 40mg,qd	141	46.0±13.0	54		
Mulder,2002	Ome 20mg, qd	151	51.6±15	88	SM I-IV	2
	Lan 30mg, qd	154	51.2±14.4	94		
	Pan 40mg, qd	156	50.8±14.5	90		
Gillessen,2004	Pan 40mg, qd	113	53±15	64	LA B-C	1 2 3
	Eso 40mg,qd	113	54±14	57		
Chen,2004	Eso 40mg,qd	52	46.3±24.7	32	NA	1 2
	Ome 20mg, qd	51	47.2±23.5	33		
Huang, 2004	Ome 20mg, qd	26	51.2	15	NA	2
	Lan 30mg, qd	26	53.6	18		

Zheng, 2004	Rab 20mg,qd	44	50.2±12.5	29	NA	2
	Pan 40mg, qd	43	51.3±10.5	28		
Fennerty,2005	Eso 40mg,qd	498	47.3 (13.2)	327	LA C-D	1 3
	Lan 30mg, qd	501	47.1 (12.9)	333		
Labenz,2005	Eso 40mg,qd	1562	50.6±14	969	LA I-IV	1 2
	Pan 40mg, qd	1589	50.5±13.8	1012		
Pace,2005	Rab 20mg,qd	277	47.7±14.2	190	SM I-III	1 2
	Ome 20mg, qd	272	47.1±14.9	184		
Schmitt,2006	Eso 40mg,qd	576	47.1±13.3	346	LA A-D	1 2 3
	Ome 20mg, qd	572	46.2±13.6	335		
Vcev,2006	Eso 40mg,qd	90	51.2±14.5	57	LA I-III	1 2
	Pan 40mg, qd	90	49.4±13.9	59		
Bardhan,2007	Pan 40mg, qd	288	53±14	141	LA I-IV	1 2 3
	Eso 40mg,qd	293	54±14	154		
Wu, 2006	Lan 30mg, qd	58	NA	NA	NA	2
	Ome 20mg, qd	56	NA	NA		
Zheng, 2006	Eso 40mg,qd	32	NA	NA	NA	2
	Ome 20mg, qd	33	NA	NA		
Cao, 2007	Eso 40mg,qd	54	46.5±20.7	30	NA	1 2
	Lan 30mg, qd	50	47.8±17.6	28		
Oyama,2008	Eso 40mg,qd	191	NA	NA	LA A-D	1 2 3
	Ome 20mg, qd	191	NA	NA		
Zheng,2009	Ome 20mg, qd	68	57.9±14.1	33	LA A-D	2
	Lan 30mg, qd	69	57.9±14.1	35		
	Pan 40mg, qd	69	57.8±13.2	34		
	Eso 40mg,qd	68	57.4±12.8	33		
Cao, 2011	Eso 40mg,qd	173	NA	NA	NA	1 2
	Ome 20mg, qd	172	NA	NA		
Song,2012	Ila 10 mg.qd	60	NA	NA	LA A-D	1 2
	Eso 40mg,qd	60	NA	NA		
Ashida,2015	Lan 30mg, qd	132	NA	NA	LA A-D	1 2 3
	Von 20mg,qd	144	NA	NA		
Ashida,2016	Von 20mg,qd	207	58.3+13.8	137	LA A-D	1 2 3
	Lan 30mg, qd	202	57.4 +13.2	154		
Xue,2016	Eso 40mg,qd	105	47.8±11.65	72	LA A-D	1 2 3
	Ila 10 mg.qd	107	48.9±12.63	75		
Yang, 2016	Ome 20mg, qd	48	NA	NA		2
	Lan 30mg, qd	48	NA	NA		
Xue,2018	Eso 40mg,qd	215	47.5±12.32	152	LA A-D	1 2
	Ila 10 mg.qd	322	48.2±11.96	232		
Uemura,2019	Von 20mg,qd	139	NA	NA	NA	2
	Lan 30mg, qd	69	NA	NA		
Li, 2019	Ila 10 mg.qd	50	47.58±3.33	23	NA	2
	Eso 40mg,qd	50	47.66±3.42	24		

Xiao,2020	Von 20mg,qd	244	54.1±13.16)	NA	LA A-D	1 2 3
	Lan 30mg, qd	237	53.8 (12.53)	NA		

1: 4 weeks healing rates 2: 8 weeks healing rates 3: adverse event rate
