# Supplementary material for: Efficacy and safety of proton pump inhibitors versus vonoprazan in treatment of erosive esophagitis: A PRISMA-compliant systematic review and network meta-analysis
Source: Medicine (Baltimore). 2022 Nov 25;101(47):e31807. doi: 10.1097/MD.0000000000031807 (PMC9704910; doi:10.1097/MD.0000000000031807)
Supplement: Supplementary file 3 [file medi-101-e31807-s003.pdf]

Table S3. Inconsistency factors of adverse event rates (previous)

Outcome	Cycle	IF and 95%CI	P
adverse event rates	Ome-Pan-Von	IF=0.414,95%CI(0.00,1.46)	0.437
	Ome-Pan-Eso	IF=0.387,95%CI(0.00,1.12)	0.302
	Ome-Lan-Eso	IF=0.173,95%CI(0.00,0.83)	0.608
	Lan-Pan-Eso	IF=0.716,95%CI(0.09,1.34) *	0.026*
	Ome-Lan-Pan	IF=0.499,95%CI(0.00,1.41)	0.284

“*” represents a significant difference.

Ome: omeprazole, 20mg/day; Pan: pantoprazole, 40mg/day; Lan: lansoprazole, 30mg/day; Rab: rabeprazole, 20mg/day; Ila: ilaprazole, 10mg/day; Eso: esomeprazole, 40mg/day; Von: vonoprazan 20mg/day ;PLA: placebo; IF= Inconsistency factor; CI= Confidence interval
