# Supplementary material for: Efficacy and safety of proton pump inhibitors versus vonoprazan in treatment of erosive esophagitis: A PRISMA-compliant systematic review and network meta-analysis
Source: Medicine (Baltimore). 2022 Nov 25;101(47):e31807. doi: 10.1097/MD.0000000000031807 (PMC9704910; doi:10.1097/MD.0000000000031807)
Supplement: Supplementary file 4 [file medi-101-e31807-s004.pdf]

Table S4. Inconsistency by node-splitting of adverse event rates (previous)

Side	Direct		Indirect		Difference		P>|z|
	Coef.	Std. Err.	Coef.	Std. Err.	Coef.	Std. Err.	
Ome-Pan	0.1663964	0.3088	-0.0617029	0.1366562	0.2280993	0.3376867	0.499
Ome-Lan	0.3430674	0.2966461	0.193965	0.1740102	0.1491023	0.3439168	0.665
Ome-Eso	-0.002319	0.1066278	0.1168117	0.2253117	-0.1191306	0.249414	0.633
Ome-Pla	0.1071446	0.3776799	0.4441354	0.3306186	-0.3369908	0.5019469	0.502
Pan-Lan *	0.6751797	0.2407436	0.0426129	0.1695838	0.6325668	0.2944759	0.032*
Pan-Eso	0.0044496	0.0899441	0.2653779	0.2209047	-0.2609283	0.2384953	0.274
Lan-Eso	0.0475058	0.1895507	-0.4552392	0.1803537	0.5027449	0.2619964	0.055
Lan-Pla	0.1786079	0.2901026	-0.1583846	0.4096228	0.3369925	0.5019462	0.502

"*" represents a significant difference.

Ome: omeprazole, 20mg/day; Pan: pantoprazole, 40mg/day; Lan: lansoprazole, 30mg/day; Rab: rabeprazole, 20mg/day; Ila: ilaprazole, 10mg/day; Eso: esomeprazole, 40mg/day; Von: vonoprazan 20mg/day ;Pla: placebo
