# Supplementary material for: Efficacy and safety of proton pump inhibitors versus vonoprazan in treatment of erosive esophagitis: A PRISMA-compliant systematic review and network meta-analysis
Source: Medicine (Baltimore). 2022 Nov 25;101(47):e31807. doi: 10.1097/MD.0000000000031807 (PMC9704910; doi:10.1097/MD.0000000000031807)
Supplement: Supplementary file 5 [file medi-101-e31807-s005.pdf]

Table S5. OR from the network meta-analysis for healing rates with a low bias risk.

OR(95%CI)							
Ome	1.17 (0.94,1.46)	1.35 (1.02,1.77)	0.78 (0.48,1.24)	1.57 (1.29,1.92)*	1.75 (1.07,2.85)*	1.56 (0.91,2.68)	0.09 (0.06,0.13)*
0.95 (0.78,1.15)	Lan	1.15 (0.86,1.54)	0.66 (0.39,1.11)	1.34 (1.08,1.67)*	1.49 (0.90,2.46)	1.33 (0.81,2.18)	0.08 (0.05,0.11)*
0.88 (0.70,1.10)	0.93 (0.73,1.18)	Pan	0.58 (0.33,0.99)*	1.17 (0.91,1.50)	1.30 (0.78,2.17)	1.16 (0.65,2.05)	0.07 (0.05,0.10)*
1.13 (0.77,1.64)	1.19 (0.78,1.82)	1.28 (0.83,1.99)	Rab	2.03 (1.22,3.39)*	2.25 (1.14,4.45)*	2.01 (0.98,4.12)	0.12 (0.06,0.21)*
0.69 (0.59,0.80)*	0.72 (0.61,0.86)	0.78 (0.64,0.95)	0.61 (0.41,0.92)	Eso	1.11 (0.71,1.74)	0.99 (0.58,1.70)	0.06 (0.04,0.08)*
0.63 (0.42,0.95)*	0.67 (0.44,1.02)	0.72 (0.47,1.10)	0.56 (0.32,0.98)	0.92 (0.63,1.35)	Ila	0.89 (0.44,1.80)	0.05 (0.03,0.09)*
0.74 (0.48,1.15)	0.78 (0.52,1.16)	0.84 (0.53,1.34)	0.66 (0.37,1.17)	1.08 (0.70,1.67)	1.17 (0.66,2.08)	Von	0.06 (0.03,0.11)*
9.36 (6.64,13.21)*	9.88 (7.03,13.89)*	10.65 (7.35,15.42)*	8.31 (4.99,13.85)*	13.65 (9.62,19.37)*	14.79 (8.84,24.73)*	12.66 (7.50,21.38)*	Pla

Results of 4 weeks healing rates were listed in right upper triangles and results of 8 weeks healing rates were listed in left lower triangles. “*” represents a significant difference.
Ome: omeprazole, 20mg/day; Pan: pantoprazole, 40mg/day; Lan: lansoprazole, 30mg/day; Rab: rabeprazole, 20mg/day; Ila: ilaprazole, 10mg/day; Eso: esomeprazole, 40mg/day; Von: vonoprazan 20mg/day ;PLA: placebo; OR= Odds Ratio; CI= Confidence interval

Table S6. The results of SUCRA with a low bias risk.

Treatment	4 weeks healing rates			8 weeks healing rates		
	SUCRA	Pr. Best	MeanRank	SUCRA	Pr.Best	MeanRank
Ome	32.3	0.0	5.7	28.8	0.0	6.0
Lan	41.1	0.0	5.1	46.0	0.0	4.8
Pan	53.8	0.1	4.2	62.4	2.4	3.6
Rab	25.0	0.4	6.3	18.3	0.2	6.7
Eso	85.1	21.6	2.0	81.9	15.3	2.3
Ila	89.0	55.2	1.8	86.3	51.8	2.0
Von	73.7	22.7	2.8	76.3	30.3	2.7
Pla	0.0	0.0	8.0	0.0	0.0	8.0

SUCRA: the surface under the cumulative ranking curve ;Pr. Best :probability of being the best; Ome: omeprazole, 20mg/day; Pan: pantoprazole, 40mg/day; Lan: lansoprazole, 30mg/day; Rab: rabeprazole, 20mg/day; Ila: ilaprazole, 10mg/day; Eso: esomeprazole, 40mg/day; Von: vonoprazan 20mg/day ;PLA: placebo
